# Pyroptosis patterns influence the clinical outcome and immune microenvironment characterization in HPV-positive head and neck squamous cell carcinoma

**DOI:** 10.1186/s13027-023-00507-w

**Published:** 2023-05-23

**Authors:** Doudou Li, Dong Ma, Yuxia Hou

**Affiliations:** 1 Key Laboratory of Shaanxi Province for Craniofacial Precision Medicine Research, College of Stomatology, Xi’an Jiaotong University, 98# XiWu Road, Xi’an, 710004 Shaanxi P.R. China; 2 Department of Orthodontics, College of Stomatology, Xi’an Jiaotong University, 98# XiWu Road, Xi’an, 710004 Shaanxi P.R. China; 3 Department of Oral and Maxillofacial Surgery, College of Stomatology, Xi’an Jiaotong University, 98# XiWu Road, Xi’an, 710004 Shaanxi P.R. China

**Keywords:** Pyroptosis, Tumor microenvironment, Prognosis, Immunotherapy, HPV-infection, Head and neck squamous cell carcinoma

## Abstract

**Background:**

Head and neck squamous cell carcinoma (HNSCC) is a heterogeneous tumor with diverse molecular pathological profiles. Recent studies have suggested the vital role of pyroptosis in tumor microenvironment. However, the expression patterns of pyroptosis in HPV-positive HNSCC are still unclear.

**Methods:**

Unsupervised clustering analysis was used to identify the pyroptosis patterns based on the RNA-sequencing data of 27 pyroptosis-related genes (PRGs) in HPV-positive HNSCC samples. Random forest classifier and artificial neural network were performed to screen the signature genes associated with pyroptosis, which were verified in two independent external cohorts and qRT-PCR experiment. Principal component analysis was used to develop a scoring system, namely Pyroscore.

**Results:**

The expression variations of 27 PRGs in HPV-positive HNSCC patients were analyzed from genomic and transcriptional domains. Two pyroptosis-related subtypes with distinct clinical outcomes, enrichment pathways and immune characteristics were identified. Next, six signature genes (GZMB, LAG3, NKG7, PRF1, GZMA and GZMH) associated with pyroptosis were selected for prognostic prediction. Further, a Pyroscore system was constructed to determine the level of pyroptosis in each patient. A low Pyroscore was featured by better survival time, increased immune cell infiltration, higher expression of immune checkpoint molecules and T cell-inflamed genes, as well as elevated mutational burden. The Pyroscore was also related to the sensitivity of chemotherapeutic agents.

**Conclusions:**

The pyroptosis-related signature genes and Pyroscore system may be reliable predictors of prognosis and serve as mediators of immune microenvironment in patients with HPV-positive HNSCC.

**Supplementary Information:**

The online version contains supplementary material available at 10.1186/s13027-023-00507-w.

## Introduction

According to the statistics in 2018, head and neck squamous cell carcinoma (HNSCC) was the 6th leading cancer worldwide, which is a heterogeneous malignancy with poor survival rates [1]. Human papillomavirus (HPV) infection is an increasingly common risk factor of HNSCC. HNSCC could be divided into three molecular subtypes: classical (CL), basal (BA) and mesenchymal (MS), whereas HPV-positive HNSCC does not fall into any group and belong to CL and MS subtypes [2]. The multi-omics data also suggest that HPV-positive HNSCC is a unique clinical entity with a specific genetic landscape and propensity for cell death compared to HPV-negative HNSCC [3]. In clinical practice, tumor immunotherapy has revolutionized traditional methods and achieved some gratifying achievements. Food and Drug Administration has recommended immune checkpoint inhibitors (ICIs) such as pembrolizumab and nivolumab as the preferred choices for patients with recurrent or metastatic HNSCC [4]. Although HPV-positive patients have been proven to be more sensitive to immune therapy, only a limited subset of patients benefited from it. Therefore, there is an urgent need to explore the tumor microenvironment of HPV-positive HNSCC to improve clinical effectiveness and targeted therapy efficiency.

The form of cell death, including necroptosis, ferroptosis and pyroptosis, has attracted more and more attention in the context of tumors. Pyroptosis, an inflammatory programmed cell death, is characterized by the cleavage of gasdermin-family perforating proteins, followed by the swelling and rupture of cell membranes, releasing intracellular contents and thus, triggering a strong inflammatory response [5]. It has been reported that pyroptosis-related genes (PRGs) were aberrantly and differentially expressed in different cancers, contributing to tumor suppression or tumor progression [6]. GSDME has been shown to enhance the phagocytosis of macrophages, as well as the number and function of NK cells and CD8(+) T lymphocytes, thereby exerting tumor inhibition [7]. Awad et al. have confirmed that NLRP1 facilitated the progression of skin cancer by mediating the expression of IL-1b and IL-18 cytokines [8]. In HNSCC, Taxol treatment has been observed to induce pyroptosis in tumor cells by mediating Caspase-1/GSDMD [9]. Zhang et al. found that the reducing expression of CD38 could prevent pyroptosis in HNSCC [10]. Notably, a series of studies have shown the close association between pyroptosis and anti-tumor immunity [11]. Given the overlapping and counteracting effects of multiple pyroptotic components, understanding the overall profile of PRGs on specific tumors, rather than the individual regulation of each component, seemed to be a more effective strategy to elucidate the crosstalk of pyroptosis and immune microenvironment in HPV-positive HNSCC.

In this paper, we comprehensively analyzed the expression profile of 27 PRGs in patients with HPV-positive HNSCC on the basis of genomic and transcriptional data. The study aimed to identify the pyroptosis-related subgroups, screen out the candidate genes and develop a pyroptosis scoring method to investigate the survival duration and immunological landscape of HPV-positive HNSCC.

## Materials and methods

### Data acquisition and processing

The analysis process of the article was presented in Additional file 1: Figure S1. Two HNSCC cohorts (TCGA-HNSCC and GSE65858) were used as the training set. The RNA-sequencing data and related clinical information of patients with HPV-positive HNSCC were retrieved from The Cancer Genome Atlas (TCGA) repository (https://portal.gdc.cancer.gov/repository) and the Gene Expression Omnibus (GEO) dataset (https://www.ncbi.nlm.nih.gov/geo/). The results for fragments per kilobase million were standardized to transcripts per kilobase million. The two datasets were combined, and the “ComBat” algorithm was used to correct batch effects. A total of 138 HPV-positive HNSCC samples and 44 normal samples with the corresponding clinical data, including overall survival (OS) time, age, gender, stage and TNM staging were enrolled. Besides, GSE3292 and GSE6792 databases from the GEO platform were combined and used as the external validation cohort. The raw data were obtained from publicly available databases and no ethical events were involved.

### Unsupervised clustering analysis of PRGs

We summarized data on 27 PRGs from previous studies [12–14]. The pyroclusters and pyroptosis-related geneclusters were identified by consensus clustering algorithm of R language ConsensuClusterPlus [15]. The selection criteria for the value of K: the modules were relatively uniformly separated and the cumulative distribution function decreased slowly [16].

### Gene set variation analysis (GSVA)

The “GSVA” package in R and a reference set (c2.cp.kegg.v7.4.symbols.gmt) retrieved from the MSigDB database were used to run the GSVA assay [17].

### Protein-protein interactions (PPI) analysis

Interactions between pyroptosis-related molecules were identified on the STRING website (https://string-db.org/).

### Identification and functional enrichment analysis of differentially expressed genes (DEGs)

The DEGs between the two pyroptosis patterns were determined by “limma” R package using the empirical Bayesian method [18]. Adjusted *p* < 0.001 and logFC = 1 were set as screening criteria. The functional enrichment analysis of DEGs, including the gene ontology (GO) terms and Kyoto Encyclopedia of Genes and Genomes (KEGG) pathways, were performed by “clusterProfiler” R package [19].

### Random forest and artificial neural network screening for signature genes

First, univariate Cox regression analysis was performed to extract significant prognostic DEGs with *p* < 0.001 as the screening criterion. Next, the prognostic DEGs were input into randomForest software package. The variable importance value was obtained by the Gini coefficient method and decreasing mean square error [20]. Genes with an importance score greater than 3 and ranked the top 6 were selected as signature genes for subsequent analysis. Then, a neural network model of the signature genes was constructed by neuralnet package [21].

### Analysis of tumor immune infiltration characteristics

The ESTIMATE algorithm was utilized to assess the immune and stromal scores and the relative purity of the tumor for each patient [22]. The CIBERSORT algorithm was utilized to calculate the proportion of 22 human immune cell subpopulations of each patient [23]. Correlations between the expression of tumor-infiltrating immune cells (TIICs) and Pyroscore were calculated using six algorithms (XCELL, TIMER, QUANTISEQ, MCPCOUNTER, EPIC and CIBERSORT-ABS algorithms) on the TIMER 2.0 platform [24]. Only differential TIICs (*p* < 0.05) were incorporated for the subsequent correlation analysis between TIICs and Pyroscore.

### Quantitative real-time polymerase chain reaction (qRT-PCR)

Five pairs of HPV-positive HNSCC and the adjacent normal tissues were collected from Stomatological Hospital of Xi’an Jiaotong University. The study was approved by the Ethical Review Committee of the Stomatological Hospital of Xi’an Jiaotong University. All patients received written informed consent. All the specimens were subjected to qRT-PCR. Total RNA was extracted from tissues according to the instructions of RNAiso Plus (Takara, Tokyo, Japan) kit. According to PrimeScript™ RT-PCR (Takara) instructions, RNA was reversely transcribed into cDNA in 20-µL volumes. The cDNA was treated by SYBR®Premix Ex Taq™II (Takara). The primers used in this experiment were shown in Additional file 2: Table S1.

### Generation of the pyroscore

We constructed a scoring system to assess the pyroptosis expression level of each patient. The procedures for establishment of Pyroscore were as follows:

First, we extracted the overlapping DEGs between the pyroptosis patterns. Then, the HPV-positive HNSCC patients were divided into several groups using an unsupervised clustering method. The consensus clustering algorithm was performed to determine the number of geneclusters as well as their stability. Next, we performed the prognostic analysis for each overlapping DEG using univariate Cox regression analysis and the genes with the significant prognosis were extracted for further analysis. Finally, the principal component analysis (PCA) was conducted to construct pyroptosis relevant score system. Both principal components 1 and 2 were selected to act as signature scores. After obtaining the prognostic value of each gene signature score, we applied a method similar to the gene expression grade index to assess the Pyroscore of each patient [25].

$${Pyroscore}_{i}=\sum (PC{1}_{i}+PC{2}_{i})$$.

Where *i* is the expression of overlapping DEGs with a significant prognosis between pyroptosis patterns.

### Correlation with drug sensitivity

Based on the Genomics of Drug Sensitivity in Cancer database (https://www.cancerrxgene.org/), we used the R package “pRRophetic” to predict the chemosensitivity of each sample. The estimated semi-inhibitory concentration (IC50) for each specific chemotherapeutic agent was obtained by the “linearRidge” function.

### Statistical analysis

Data processing was completed by using the PERL language program (version 5.32.1). Statistical analyses were performed utilizing R software and related calculate packages (version 4.1.1). For clusters of two normally distributed variables, unpaired Student’s t-tests were used for analysis. For clusters of two non-normally distributed variables, the Wilcoxon rank-sum test was used to analyze. For multiple comparisons, Kruskal-Wallis and one-way ANOVA tests were used. Correlations between the variables were analyzed using Spearman’s correlation coefficient. The “Surv-cut point” function in the R package ‘Survminer’ was used to evaluate the cutoff point of each group. The “Surv-cut point” function was based on maximally selected rank statistics, which enabled the fast assessment of all potential cut points, to find the separating partition and determine the best cut-off point of continuous variables [26]. Survival curves were generated using the Kaplan-Meier (K-M) method and the log-rank test was used to determine differences between groups. If not special explanation above, *p* < 0.05 was deemed as statistically significant and all tests were two-sides.

## Results

### Overview of genetic and transcriptional variations of PRGs in HPV-positive HNSCC

**Fig. 1:**
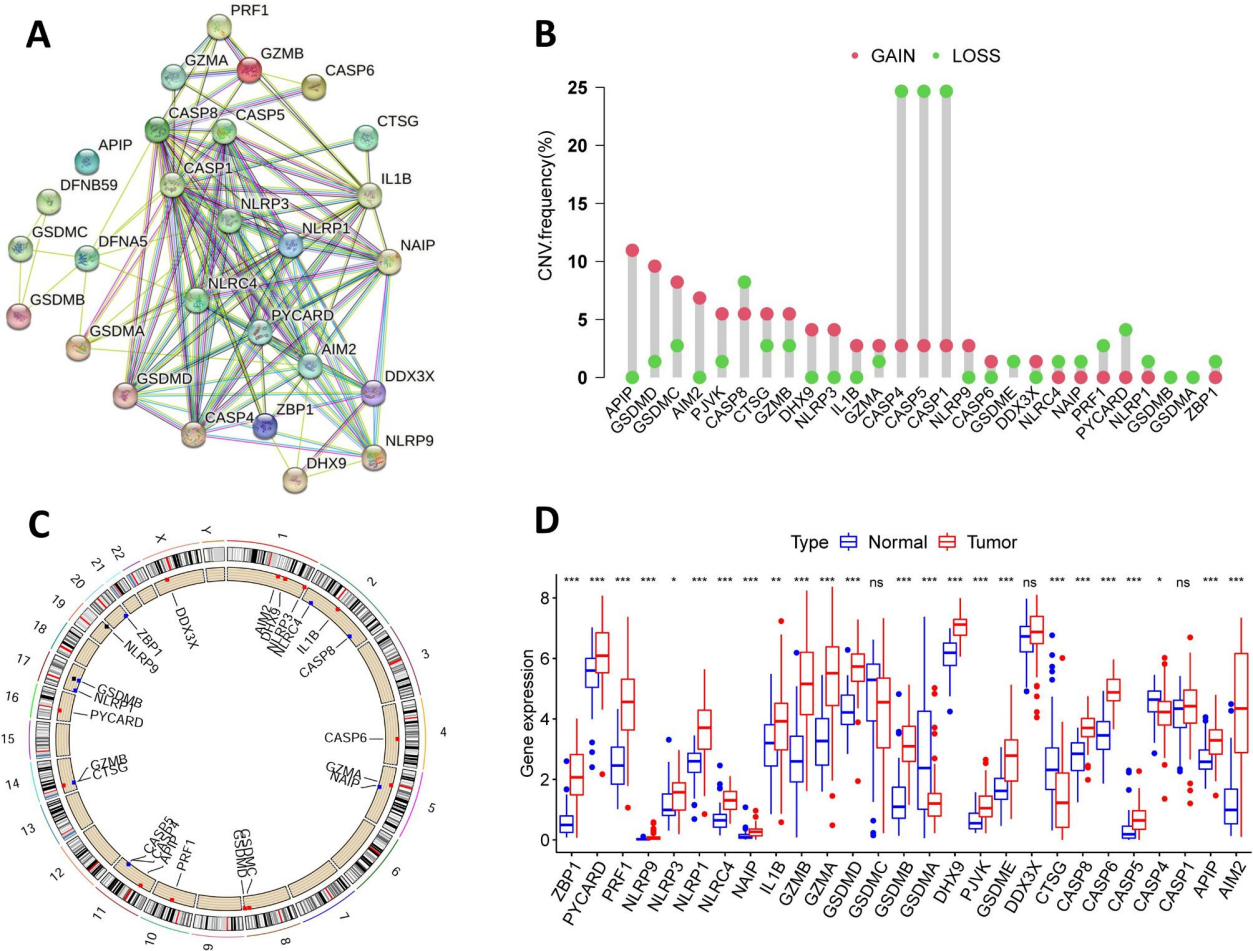
Genomic and transcriptional variations of pyroptosis-related genes (PRGs) in HPV-positive HNSCC. (**A**) The potential protein-protein interaction networks among 27 pyroptosis-related proteins were drawn on the STRING website. (**B**) Frequency of copy number variations (CNV) gain and loss in 27 PRGs. (**C**) The chromosomal localization information of PRGs with CNV. (**D**) Expression levels of 27 PRGs in HPV-positive HNSCC and normal tissues. *p < 0.05, **p < 0.01, and ***p < 0.001

In this study, protein-protein interaction networks functional enrichment analysis of the 27 pyroptosis-related molecules were analyzed by STRING platform, which showed widespread protein interactions of PRGs (Fig. 1A). Next, somatic copy number variation (CNV) of these PRGs in HPV-positive HNSCC were analyzed (Fig. 1B). Among them, the CNV of APIP, GSDMD, GSDMC and AIM2 significantly increased, while the CNV of CASP4, CASP5 and CASP1 remarkably decreased. Figure 1 C has shown the chromosomal localization information of these PRGs with CNV. In addition, we compared PRGs expression in normal and HPV-positive HNSCC tissues. As shown in Fig. 1D, the majority of PRGs were significantly elevated in tumor tissue, while GSDMA and CTSG were significantly decreased. Collectively, the above results have shown the genomic and transcription profiles of PRGs and their significant differences between normal and HPV-positive HNSCC tissues, suggesting the imbalance of PRGs and its potential role in the occurrence of HPV-positive HNSCC.

### Identification of two different pyroptosis subtypes

**Fig. 2:**
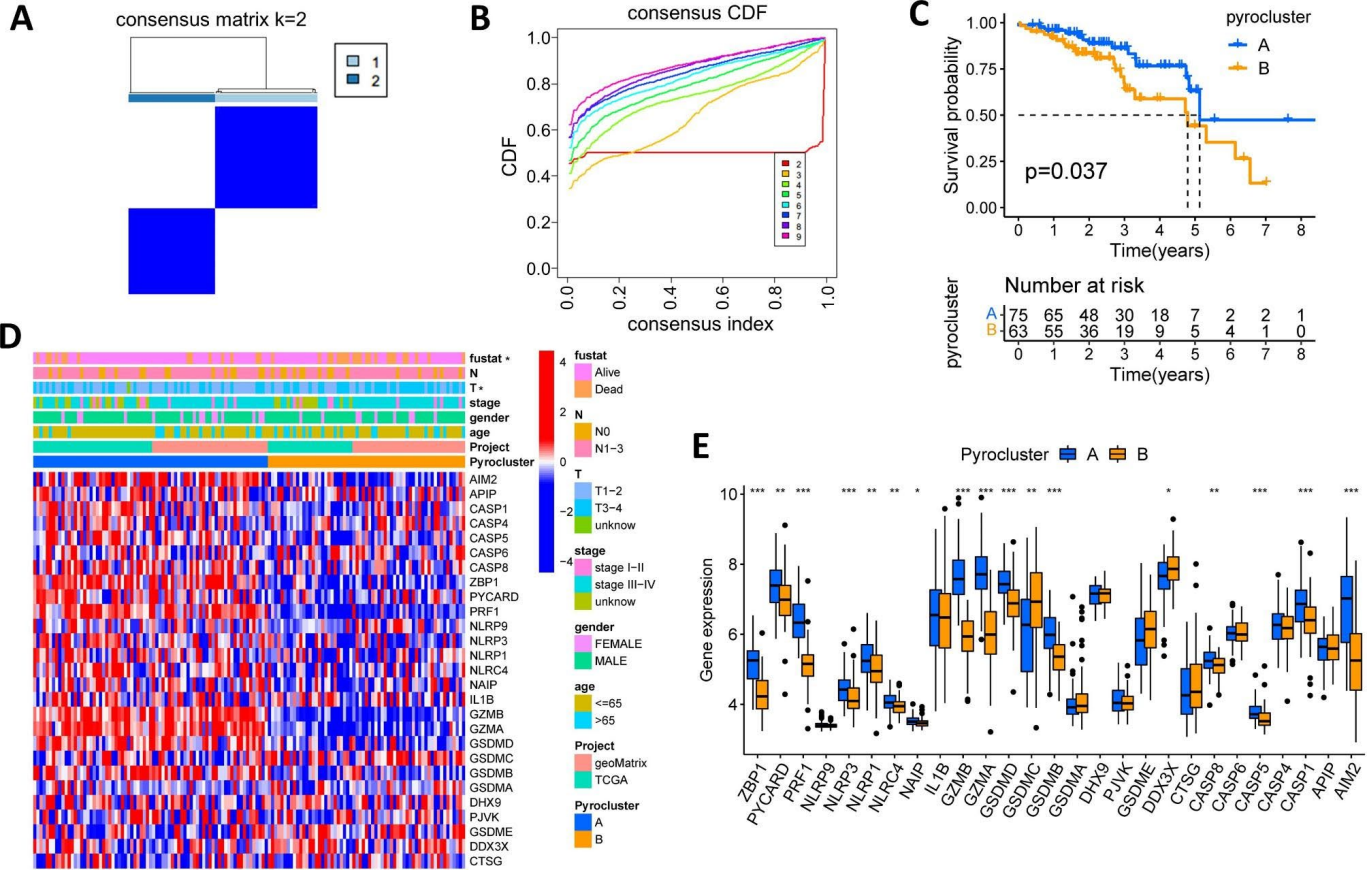
Unsupervised learning to identify two pyroclusters based on the expression of 27 PRGs. (**A**) Consensus clustering matrix defining two patterns (k = 2) in TCGA-HNSCC and GSE65858 cohorts. (**B**) The cumulative distribution function of clustering. (**C**) The two pyroclusters have a substantial difference in overall survival time according to Kaplan-Meier survival analysis. (**D**) The heatmap shows the clinicopathological traits and unsupervised clustering of the 27 PRGs in TCGA-HNSCC and GSE65858 cohorts. Red denotes higher expression, whereas blue denotes lower expression. (**E**) Relative expression levels of 27 PRGs between the two pyroclusters

To reveal the expression patterns of pyroptosis in HPV-positive HNSCC. The RNA expression of 138 HPV-positive HNSCC patients from TCGA-HNSCC and GSE65858 databases was extracted. With the unsupervised clustering algorithm in R language, we found that when the samples were clustered into two modules (k = 2), the clustering stability achieved satisfactory results (Fig. 2A and B and Additional file 1: Figure S2). The tumor samples were divided into two types, namely pyrocluster A (n = 66) and pyrocluster B (n = 72). K-M analysis showed that patients in pyrocluster A had a significantly better OS time than those in pyrocluster B (*p* = 0.037, Fig. 2C). Then, we examined the association between clinicopathological features and scorch molecule expressions in the two pyroclusters. As shown in Fig. 2D, pyrocluster B had significantly higher mortality and lower T staging (*p* < 0.05) compared to pyrocluster A. The majority of PRGs was significantly higher in pyrocluster A than that in pyrocluster B (Fig. 2E).

### The two pyroptosis patterns associated with distinct biological processes and immune infiltration characteristics

**Fig. 3:**
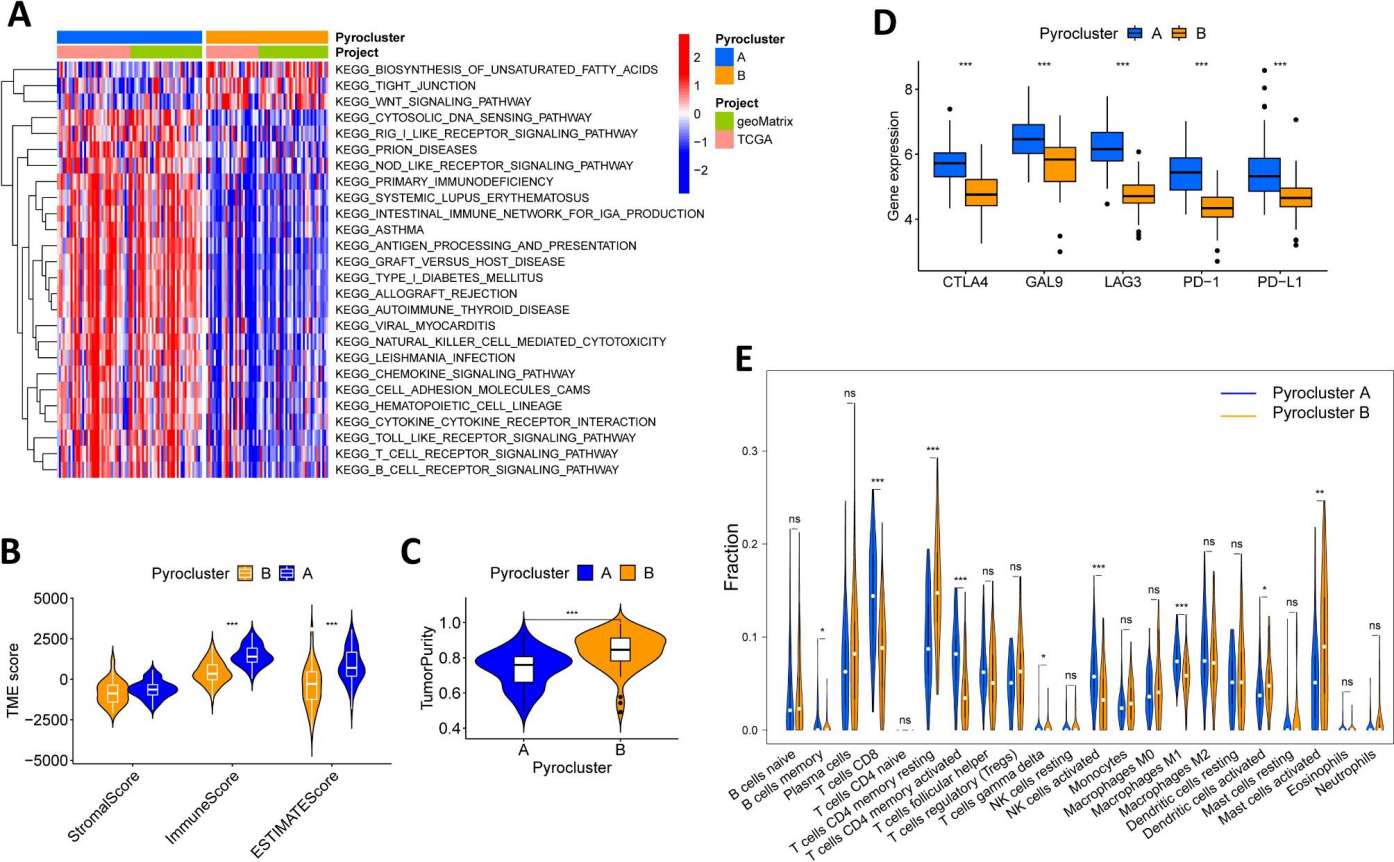
Biological processes and immune characteristics of the two pyroclusters. (**A**) The heatmap shows the biological processes in the two pyroclusters analyzed by GSVA. Red denotes activated pathways and blue denotes inhibited pathways. (**B-C**) Tumor immune microenvironment scores (**B**) and tumor purity (**C**) between the pyroclusters. (**D**) The expression of immune checkpoint molecules between the two pyroclusters. (**E**) The abundance of 22 tumor immune infiltrating cells between the two pyroclusters

Then, we analyzed the enrichment pathways of the two patterns to explore the underlying biological processes of pyroptosis in HPV-positive HNSCC. The GSVA enrichment analysis showed the differential enrichment pathways between the two pyroclusters. As the results suggested, pyrocluster A was enriched in immune-activated hallmarks such as Toll-like receptor signaling pathway, NOD-like receptor signaling pathway, T and B cell receptor signaling pathways (Fig. 3A and Additional file 2: Table S2). To further understand the influence of pyroptosis-related molecules on TME in HPV-positive HNSCC patients, we utilized the ESTIMATE algorithm to assess the immune and stromal scores as well as the relative tumor purity of each patient. We found that patients in pyrocluster A had remarkably higher immunescores and estimatescores, while patients in pyrocluster B had remarkably higher tumor purity (Fig. 3B C). Given the unique role of immune checkpoint molecules in tumor immune microenvironment (TIME), we explored the expression of PD-1, PD-L1, LAG3, GAL9 and CTLA4 and found that the expression of these molecules was significantly elevated in pyrocluster A than that in pyrocluster B (Fig. 3D). Moreover, we estimated the infiltration characteristics of 22 immune cells in pyroptosis patterns using the CIBERSORT algorithm. The results showed that the activated TIICs, especially B memory cells, CD8(+) T cells, CD4 memory-activated T cells, activated NK cells, and M1 macrophage cells infiltration were notably higher in pyrocluster A than those in pyrocluster B, while CD4 memory-resting T cells, regulatory T cells (Tregs), activated dendritic cells and activated mast cells infiltration were notably higher in pyrocluster B than those in pyrocluster A (Fig. 3E).

### Two scorch gene subtypes were recognized using prognostic DEGs

**Fig. 4:**
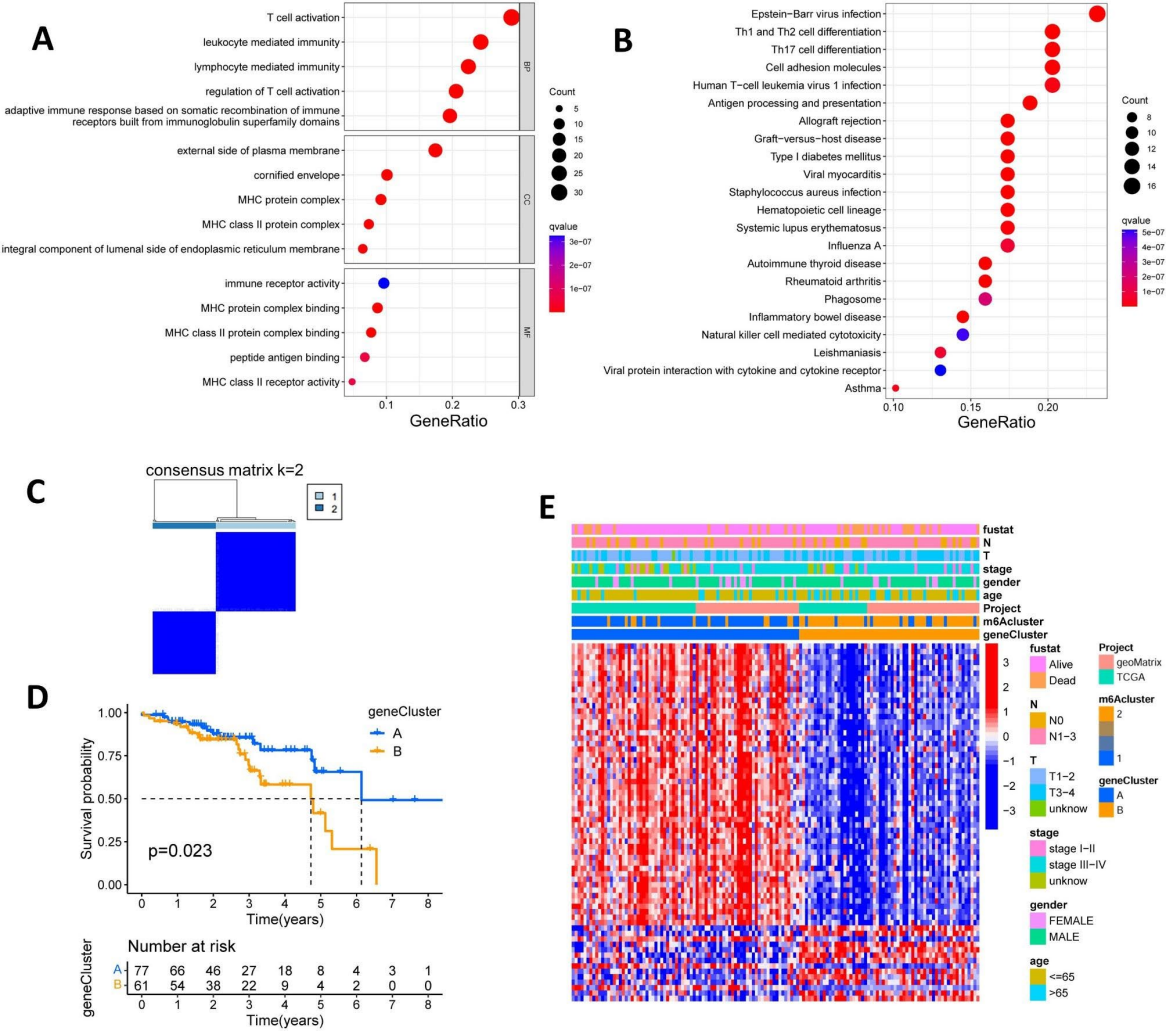
Generation of three pyroptosis-related geneclusters based on prognostic differentially expressed genes (DEGs). (**A-B**) Gene Ontology (A) and Kyoto Encyclopedia of Genes and Genomes (**B**) enrichment analysis of DEGs between the two pyroclusters. (**C**) Consensus clustering matrix defining two genomic subtypes (k = 2). (**D**) Kaplan-Meier survival analysis shows significant differences in overall survival time between genecluster A and genecluster B. (**E**) The heatmap shows the correlation between clinicopathological traits and the geneclusters. Red denotes high expression and blue denotes low expression

By conducting the “limma” package, the DEGs between the two pyroptosis patterns were identified. To explore the biological mechanisms involved in pyroptosis-related molecules, functional enrichment analysis of DEGs was performed. GO analysis exhibited the top 5 cellular components, molecular functions and biological processes respectively, revealing that immune-related processes, especially major histocompatibility complex (MHC) proteins and T-cell were activated (Fig. 4A and Additional file 2: Table S3). The antigen processing and presentation process by MHC II affected T cell recognition and tumor cell killing [27]. KEGG analysis indicated that the DEGs were mainly enriched in immune and inflammation related pathways, such as Th1 and Th2 cell differentiation, Th17 cell differentiation and inflammatory bowel disease (Fig. 4B and Additional file 2: Table S3).

Next, the univariate Cox regression analysis was performed to screen out the prognosis related DEGs. With *p* < 0.001 as the screening criterion, a total of 66 prognostic DEGs between the two scorch patterns were identified (Additional file 2: Table S4). Consequently, 138 HNSCC HPV-positive patients were classified into 2 genomic subtypes using the consensus clustering algorithm based on the expression of the 66 DEGs (Fig. 4C and Additional file 1: Figure S3). K-M analysis method showed the significant difference of OS time between the gene subtypes (*p* = 0.023) (Fig. 4D). The heatmap showed the expression of the 66 genes in the geneclusters and the pyroptosis patterns as well as the relationship between these genes and clinical parameters (Fig. 4E). In addition, the two geneclusters exhibited significant differences in PRGs expression (Additional file 1: Figure S4).

### Random forest and neural network algorithms to explore the signature genes

**Fig. 5:**
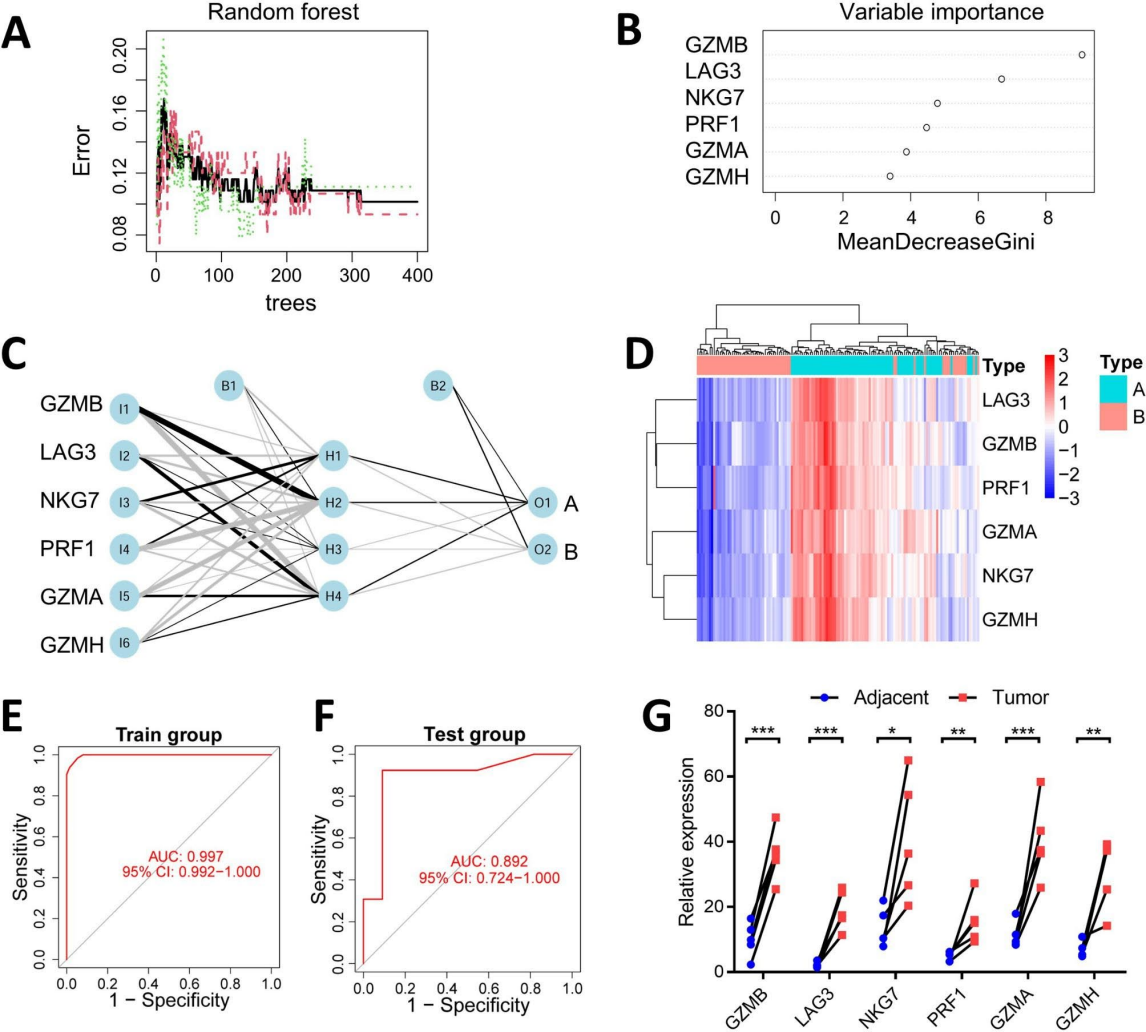
Exploration of the signature genes by random forest classifier and neural network model. (**A**) The relationship plot between the error rate and the number of decision trees. When the number of decision trees is about 400, the error rate is relatively stable. (**B**) The importance score of the signature genes using the Gini coefficient method. The X-axis denotes the signature genes, and the Y-axis denotes the importance index. (**C**) The neural network diagram demonstrates the relationship between the signature genes and the two pyroclusters. (**D**) The signature genes were differentially expressed in the two pyroclusters. (**E-F**) The receiver operating characteristic curve analysis of the classification efficiency of the signature genes in training sets (TCGA-HNSCC and GSE65858 cohorts) and external testing sets (GSE3292 and GSE6792 cohorts). (**G**) Expression levels of the 6 signature genes in HPV-positive HNSCC and their adjacent normal tissues using qRT-PCR.

To find the signature genes associated with the pyroptosis, we utilized the random forest algorithm to assess the importance of 66 prognostic DEGs (Fig. 5A). The variable importance was measured by the Gini coefficient method and we defined genes with an importance score greater than 3 and ranked the top 6 as the signature genes, including GZMB, LAG3, NKG7, PRF1, GZMA and GZMH (Fig. 5B). Consequently, the artificial neural network model depicting the distribution of the signature genes in pyroclusters was shown in Fig. 5C. The weights of the signature genes were shown in Additional file 2: Table S5. The heatmap showed the signature genes could distinguish the two pyroclusters (Fig. 5D). It could be seen that all signature genes were low expressed in pyrocluster A, whereas high expressed in pyrocluster B. Then, the classification efficiency of the model was evaluated by receiver operating characteristic (ROC) curves in the training group (TCGA-HNSCC and GSE65858 cohorts). As shown in Fig. 5E, the area under the curve (AUC) of the training group was up to 0.997. The performance was confirmed in the external GEO datasets (GSE3292 and GSE6792 cohorts). As shown in Fig. 5F, the AUC of the test group was up to 0.892. In addition, the mRNA expression levels of the signature genes in HPV-positive HNSCC tissues were verified to be higher than that in adjacent normal tissues detected by qRT-PCR (Fig. 5G).

### Construction and validation of the prognostic pyroscore

**Fig. 6:**
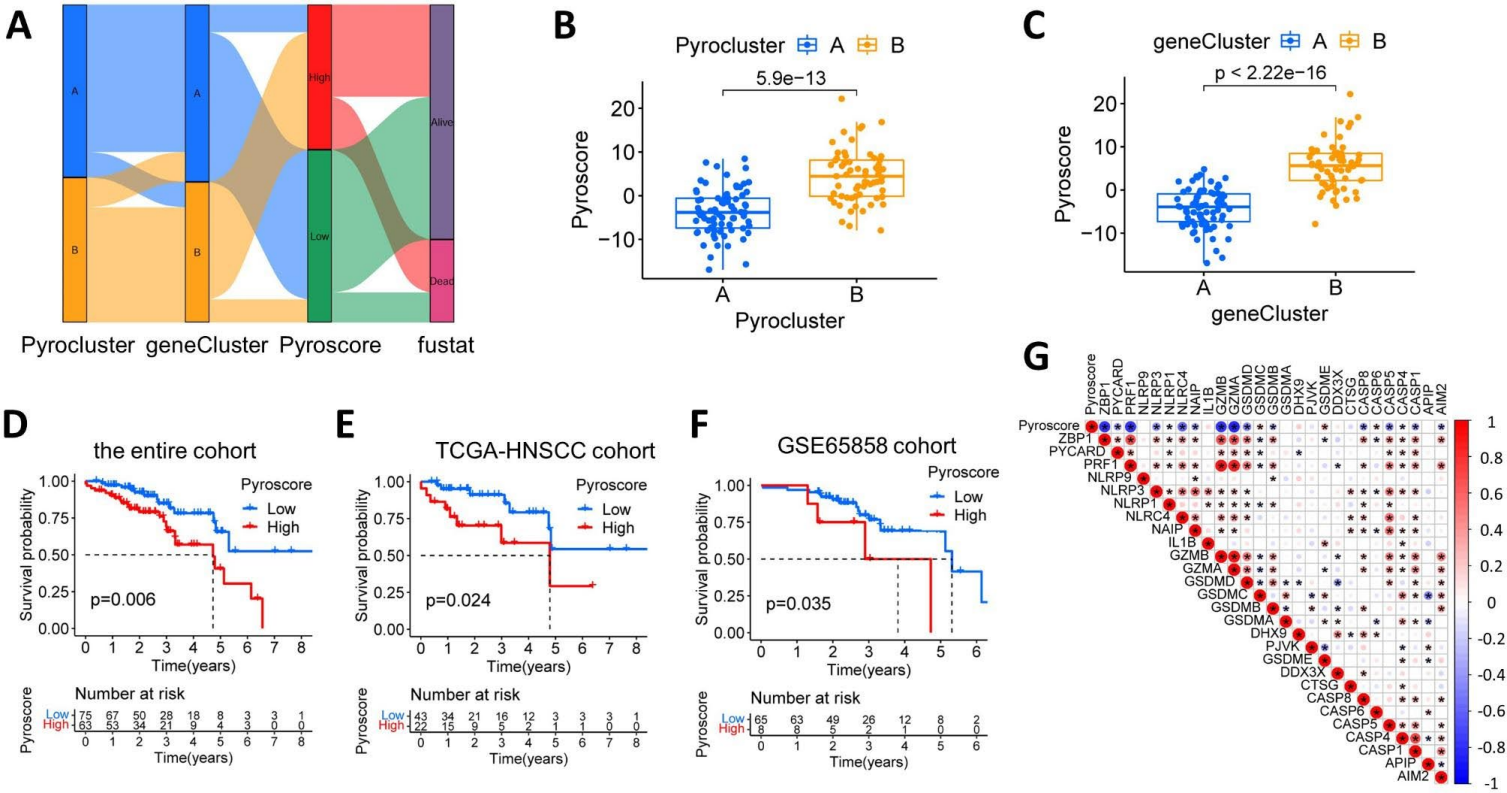
Construction and validation of Pyroscore. (**A**) Alluvial diagram showing the distributions of pyroclusters, geneclusters, Pyroscores and survival outcomes. (**B**) Pyroscore is differentially expressed in pyrocluster A and B. (**C**) Pyroscore is differentially expressed in genecluster A and B. (**D**) Kaplan-Meier survival analysis shows a significant difference in overall survival time between high and low Pyroscore groups in training sets (TCGA-HNSCC and GSE65858 cohorts). (**E**) Kaplan-Meier analysis of overall survival time between high and low Pyroscore groups in internal testing set (TCGA-HNSCC cohort). (**F**) Kaplan-Meier analysis of overall survival time between high and low Pyroscore groups in internal testing set (GSE65858 cohort). (**G**) The correlations among Pyroscore and PRGs. Red dots denote positive correlations and blue dots denote negative correlations. Larger and deeper dots represent stronger correlations

The above studies demonstrated a robust association among pyroptosis, tumor immunity and survival in HNSCC HPV-positive patients, so we introduced a unique scoring scheme, the Pyroscore, to assess the pyroptosis expression level of each patient. By employing principal component analysis, the sum of the top two contributing components was used as the Pyroscore for each patient. By conducting the R package “Survminer”, the optimal cut-off was calculated and then patients were divided into high Pyroscore and low Pyroscore groups. As shown in Fig. 6A, the distributions of genecluster, pyrocluster, Pyroscore, and survival status were plotted as a Sankey diagram. Of note, Pyroscore was confirmed to be effective in evaluating the pyroclusters and geneclusters. Comparing the level of scorch death among the subtypes, it was found that pyrocluster A and genecluster A had notably lower Pyroscore, while pyrocluster B and genecluster B had notably higher Pyroscore (Fig. 6B C). In addition, in the training and two validation cohorts, K-M analysis showed significantly higher OS time in low Pyroscore groups than those in high groups (*p* = 0.006; *p* = 0.024; *p* = 0.035;Fig. 6D - F). Moreover, Pyroscore was found to be negatively associated with the expression of most PRGs, especially GZMA, GZMB and PRF1 (Fig. 6G). Overall, Pyrocluster A and genecluster A with lower Pyroscore showed better OS time, which was consistent with our previous results.

### Pyroscore associated with immune cell infiltration and TME

**Fig. 7:**
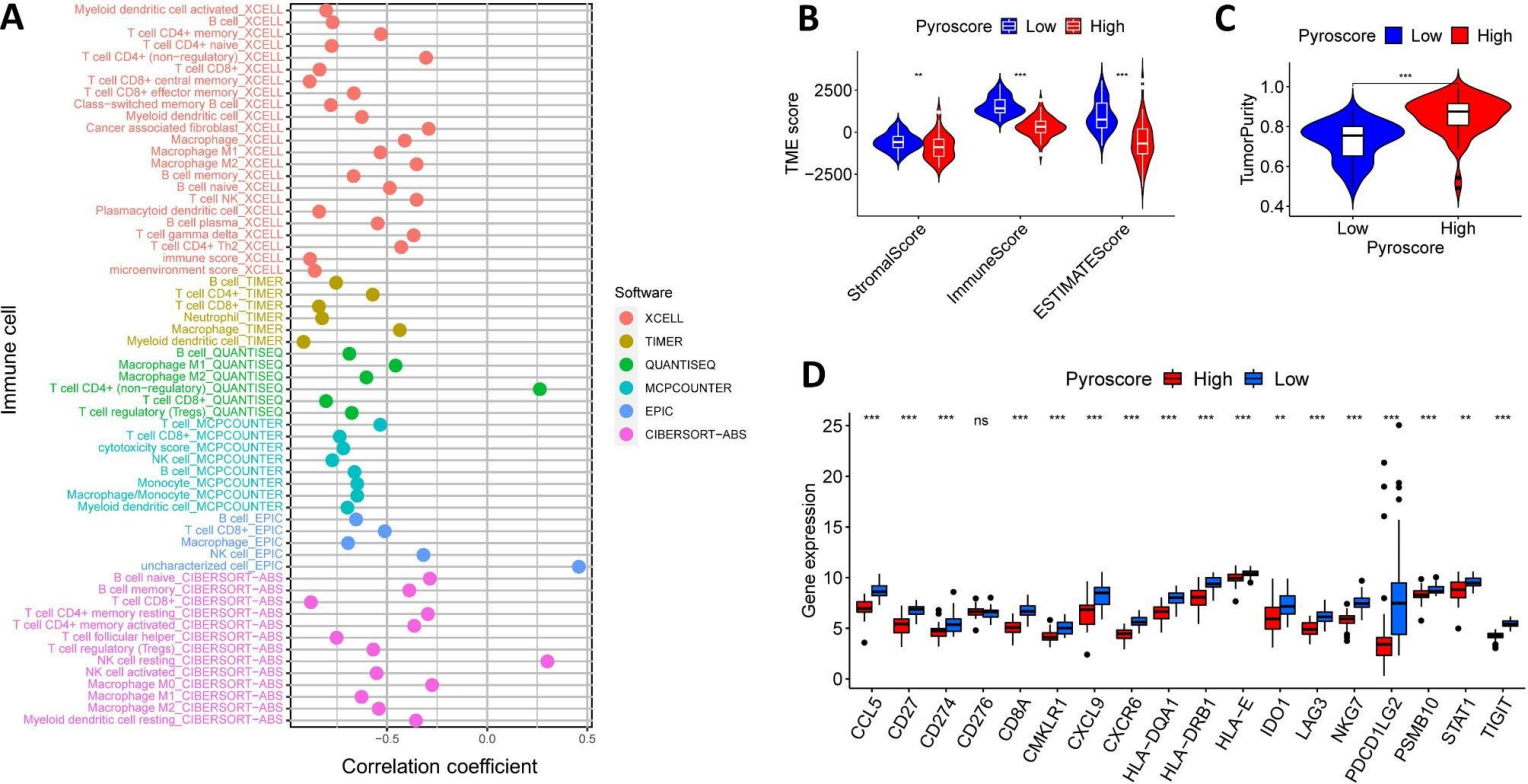
The immune characteristics of different Pyroscore subgroups. (**A**) Bubble plot depicting the correlation between Pyroscore and immune infiltrating cells. (**B-C**) Tumor immune microenvironment scores (**B**) and tumor purity (**C**) in different Pyroscore subgroups. (D) The expression profile of 18 T cell-inflamed genes in different Pyroscore subgroups

The abundance of immune cells was thoroughly examined using XCELL, TIMER, QUANTISEQ, MCPCOUNTER, EPIC and CIBERSORT-ABS algorithms. Surprisingly, Pyroscore was negatively correlated with the level of infiltration of most immune cells, except for uncharacterized cells (EPIC) and NK-resting cells (CIBERSORT-ABS) (Fig. 7A and Additional file 2: Table S6). Of note, CD 8(+) T cell was strongly correlated with Pyroscore through the detection by different platforms. Then, we used the ESTIMATE algorithm to evaluate the immune and matrix components in the TME. Patients in the low Pyroscore group had higher stromalscore, immunescore and ESTIMATEscore as well as lower tumor purity compared to the high Pyroscore group (Fig. 7B C). In addition, T cell–inflamed gene expression profile (GEP) is a symbol of CD 8(+) T cell activation and closely reflects the tumor’s sensitivity to immune response [28]. Of the 18 T cell-inflamed genes, 17 genes were considerably higher in the low Pyroscore groups than that in the high Pyroscore groups (Fig. 7D).

### Clinical implications of pyroscore in antitumor therapy

**Fig. 8:**
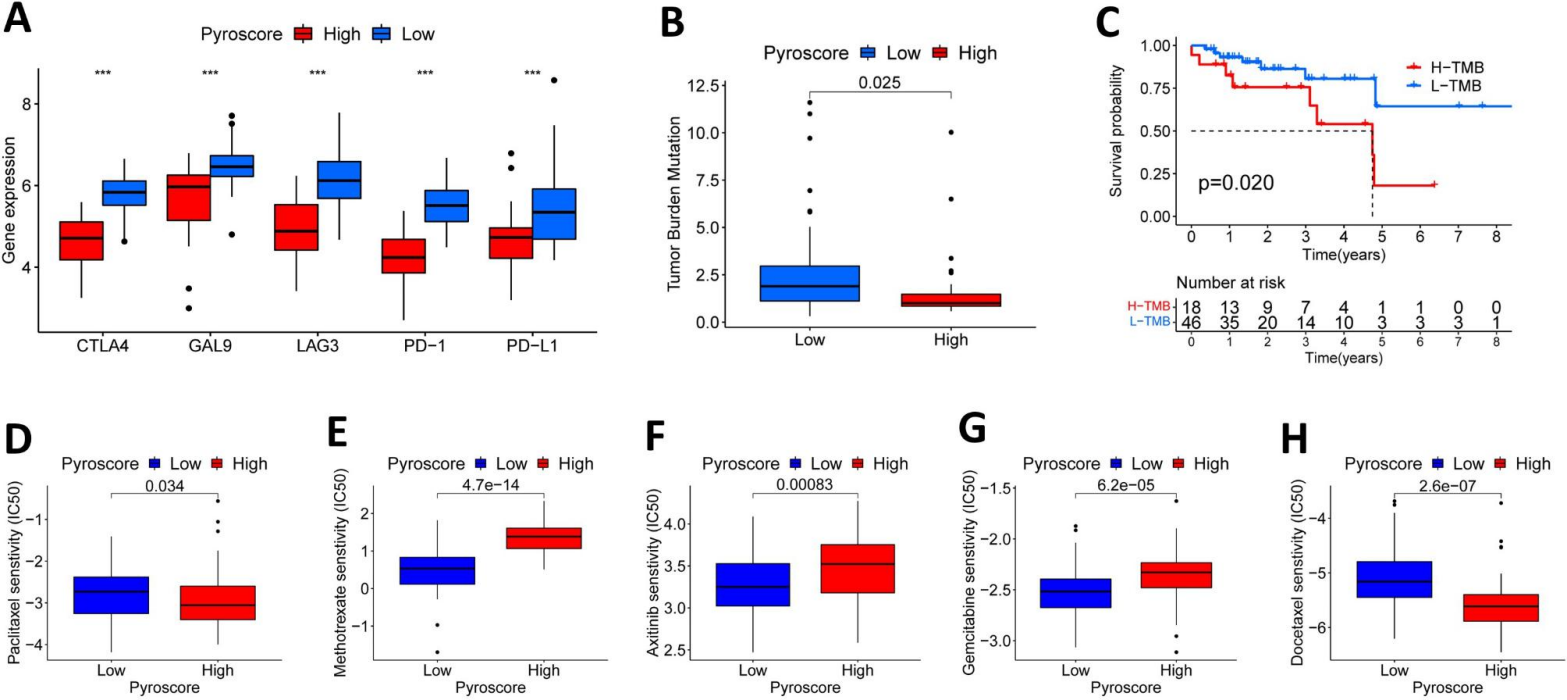
The role of Pyroscore in anti-tumor therapy. (**A**) The expression of immune checkpoint molecules in Pyroscore groups. (**B**) The tumor mutational burden in Pyroscore groups. (**C**) Kaplan-Meier survival analysis shows significant differences in overall survival time between high and low Pyroscore groups. (**D-G**) The sensitivity of commonly used drugs is significantly different in Pyroscore groups

Considering the critical value of immune checkpoints in immunotherapy, we compared the expression levels of CTAL-4, GAL9, LAG3, PD-1 and PD-L1 in Pyroscore groups. We found that the low Pyroscore group had higher expression of CTAL-4, GAL9, LAG3, PD-1and PD-L1, suggesting that patients with low Pyroscore may be more sensitive to immunosuppressive therapies (Fig. 8A). In addition, studies have shown that tumor mutational burden (TMB) may assist in predicting clinical response, which may be associated with neoantigens generated by the mutation, thereby enhancing the immune system’s ability to recognize cancer cells [29]. As shown in Fig. 8B, we found that patients with low Pyroscore showed a significantly higher TMB than patients with high Pyroscore. Further, compared with the high TMB group, patients with low TMB have a better OS time (Fig. 8C). We next selected the commonly used chemotherapeutic agents for the treatment of HNSCC in order to examine drug sensitivity variations between patients with high and low Pyroscores. To compute IC50 drug sensitivity values, we utilized the R package ‘pRRophetic.‘ The IC50 values of paclitaxel and docetaxel were considerably lower in the high Pyroscore group, whereas the values of axitinib, methotrexate, and gemcitabine were significantly higher in the high Pyroscore group compared to the low Pyroscore group (Fig. 8D-H).

## Discussion

The intense inflammatory response elicited by pyroptosis was confirmed to affect TIME and tumor progression [30, 31]. Although immunotherapy has made great strides in HNSCC, tumor heterogeneity has yielded unpredictable clinical responses, which was a serious challenge for antitumor therapy. HPV infection has been reported to encompass much of the heterogeneity of HNSCC [2]. Therefore, we focused on the pyroptosis patterns in HPV-positive HNSCC to better understand and manage this disease.

In this study, we firstly explored genetic and transcriptional variations of 27 PRGs in HPV-positive HNSCC and found that the most expression of PRGs was significantly increased in tumor samples than that in normal samples. Then, two pyroptosis subtypes were identified based on the expression of PRGs. Surprisingly, the two pyroptosis patterns exhibited distinct prognosis, biological processes and TIME characteristics. Compared with pyrocluster B, pyrocluster A had significantly better survival time. Besides, pyrocluster A was significantly associated with more enriched immune pathways, higher immune cell abundance, higher expression of immune checkpoint molecules and activated TIICs, which indicated that pyrocluster A was an immune “hot” tumor compared to pyrocluster B. A “hot” tumor indicated a better treatment response to immunotherapy. Studies also have reported that pyroptosis inducers combined with PD-1 inhibitors could turn a “cold” tumor into a “hot” tumor that the immune system could recognize, which demonstrated the enormous therapeutic application value of pyroptosis [32].

Next, we explored the pyroptosis subtypes related genes and found that the inflammatory and immune-related biological processes were involved, especially the activation of MHC proteins and T-cells. Based on these pyroptosis-related genes, six signature genes associated with pyroptosis (GZMB, LAG3, NKG7, PRF1, GZMA and GZMH) were identified and their prognostic value was verified. Given the importance of pyroptosis in prognosis and TIME, we developed a novel scoring system, Pyroscore, to quantify pyroptosis levels in patients with HPV-positive HNSCC. Herein, patients with low Pyroscores had significantly better OS time than those with high Pyroscores. Besides, Pyroscore was negatively associated with the expression of most PRGs, especially GZMA, GZMB and PRF1. Previous reports have shown that when GZMA cleaved GSDMB or GZMB cleaved GSDME, NK cells and CD8(+) T cells directly triggered pyroptosis of tumor cells [7, 32]. The killing of tumor cells by NK cells and cytotoxic T lymphocytes was the ultimate pivotal event of antitumor immunity and was previously thought to be non-inflammatory. In GSDM-expressing tumors, the killing effect of immune cells could be transformed into inflammatory pyroptosis when gasdermin was directly cleaved and activated by granzymes [33]. It was proved that GSDME-mediated pyroptosis induced by chemotherapy drugs played a role in anti-tumor response of oral cancer [34]. Recently, PRF1 has been reported to be a prognostic marker with implications on the immune infiltration of HNSCC [35]. Our findings have suggested the feasibility of targeted pyroptosis to aid the clinical treatment for patients with HNSCC.

TIME refers to the milieu in which tumor cells live, which includes blood vessels, extracellular matrix, immune cells, and signaling molecules, etc [36]. The occurrence and development of tumor is the result of the interaction between tumor cells and their microenvironment. TIICs are closely associated with immunotherapy sensitivity and understanding the composition of immune cells in TIME can reveal the heterogeneity of tumors [37]. In this study, pyrocluster A associated with low Pyroscore was an actived immune signature. Numerous evidence has reported that effector and memory T cells exert tumor-killing functions, whereas Tregs weaken anti-tumor immune responses and exert immunosuppressive effects [38]. Herein, pyrocluster A was infiltrated with CD8(+) T cells and CD4 memory-activated T cells, while pyrocluster B showed higher infiltration of CD4 memory-resting T cells and Tregs. Besides, Pyroscore was also strongly correlated with CD8(+) T cells. M1-type macrophages exerted pro-inflammatory and anti-tumor effects, but tumor-associated macrophages in TIME were M2-type and promoted angiogenesis and tumor invasion by secreting Th2 cytokines [39]. Pyrocluster A had higher M1 macrophage cells infiltration than pyrocluster B. Recent studies revealed that B cells were the strongest prognostic element and involved in the immune response [40]. In this study, pyrocluster A with better survival time had higher infiltration of B cells, which was consistent with the previous studies.

Tumor cell resistance to the immunosuppressive microenvironment, such as through PD-1, is a major dilemma in antitumor therapy. Blocking immune checkpoint proteins through ICIs can reawaken the immune system. However, once activated, T cells must be sufficient to distinguish tumors from normal cells. The presence of immunogenic neoantigens on the surface of cancer cells helps the immune system to recognize cancer cells in the context of MHC [41]. Due to the increased neoantigens produced by somatic tumor mutations, the higher the number of mutations (higher the TMB), the higher the likelihood that certain neoantigens presented by MHC proteins are immunogenic and thus can help T cells to recognize and eradicate cancer cells [29]. Besides, studies have demonstrated that T cell-inflamed GEP was a unique feature of T-cell activation and could manifest clinical antitumor efficacy [28]. The high T cell-inflamed GEP was associated with a significant pan-cancer survival benefit and favored anti-PD-1 immunotherapy [42]. Patients in pyrocluster A with low Pyroscore had higher TMB, elevated expression of immune checkpoint molecules (CTAL-4, GAL9, LAG3, PD-1 and PD-L1) and T cell–inflamed genes, which indicates a better response to immunotherapy, especially to ICIs treatment. In addition, patients in high and low Pyroscore groups had different sensitivities to commonly used chemotherapeutic agents, suggesting that Pyroscore could assist in guiding clinical drug administration.

However, there are some limitations in this study. First, we did not find clinical data on HPV-positive HNSCC patients treated with ICIs to further validate our results. Second, the original data used in this study is retrospective from the cross-queue, and prospective studies are necessary to avoid the inherent errors of retrospective research in the future.

## Conclusions

The need for personalized treatment of patients has been proposed because of the heterogeneity of tumors. We comprehensively analyzed the pyroptosis patterns of HPV-positive HNSCC and revealed the underlying biological processes and immune infiltration characteristics affected by the PRGs. Besides, the pyroptosis-related signature genes and the Pyroscore system were developed to explore the feasibility of targeted pyroptosis assisted immunotherapy for HPV-positive HNSCC. The landscape of pyroptosis facilitates our understanding of the TIME and lays the foundation to improve the immunotherapeutic efficacy of HNSCC.

## Electronic supplementary material

Below is the link to the electronic supplementary material.

**Additional file 1**:
**Figure S1** The workflow of the study.
**Figure S2** Clustering of pyroclusters and consensus matrix heatmaps for k = 3–9.
**Figure S3** Unsupervised clustering of pyroptosis-related geneclusters and consensus matrix heatmaps for k = 3–9.
**Figure S4** Relative expression levels of 27 PRGs between the two geneclusters. *p < 0.05, **p < 0.01, and ***p < 0.001.

**Additional file 2**:
**Table S1** The primer sequences for qRT-PCR.
**Table S2** The activation states of biological pathways in pyroptosis subtypes by GSVA enrichment analysis.
**Table S3** Functional annotation of the differentially expressed genes between the two pyroptosis subtypes.
**Table S4** Prognostic analysis of 66 pyroptosis-related genes using a univariate Cox regression analysis.
**Table S5** The output results of the neural network model.
**Table S6** Correlation between tumor-infiltrating immune cells and Pyroscore.

## Data Availability

The raw data in this study could be downloaded from the following databases: (https://portal.gdc.cancer.gov/repository and https://www.ncbi.nlm.nih.gov/geo/). The relevant codes are available from the corresponding author on reasonable requests.

## Abbreviations

AUC: Area under the curve
CNV: Copy number variation
DEGs: Differentially expressed genes
GEO: Gene Expression Omnibus
GEP: Gene expression profile
GO: Gene ontology
GSVA: Gene set variation analysis
HNSCC: Head and neck squamous cell carcinoma
HPV: Human papillomavirus
ICIs: Immune checkpoint inhibitors
K-M: Kaplan-Meier
KEGG: Kyoto Encyclopedia of Genes and Genomes
MHC: Major histocompatibility complex
OS: Overall survival
PPI: Protein-protein interactions
PRGs: Pyroptosis-related genes
qRT-PCR: Quantitative real-time polymerase chain reaction
TCGA: The Cancer Genome Atlas
TIICs: Tumor-infiltrating immune cells
TIME: Tumor immune microenvironment
TMB: Tumor mutational burden
