# Supplementary material for: Pyroptosis patterns influence the clinical outcome and immune microenvironment characterization in HPV-positive head and neck squamous cell carcinoma
Source: Infect Agent Cancer. 2023 May 23;18:30. doi: 10.1186/s13027-023-00507-w (PMC10207749; doi:10.1186/s13027-023-00507-w)
Supplement: Supplementary file 1 — Additional file 1: Figure S1 The workflow of the study. Figure S2 Clustering of pyroclusters and consensus matrix heatmaps for k = 3–9. Figure S3 Unsupervised clustering of pyroptosis-related geneclusters and consensus matrix heatmaps for k = 3–9. Figure S4 Relative expression levels of 27 PRGs between the two geneclusters. *p < 0.05, **p < 0.01, and ***p < 0.001. [file 13027_2023_507_MOESM1_ESM.docx]

**Supplementary Figures**


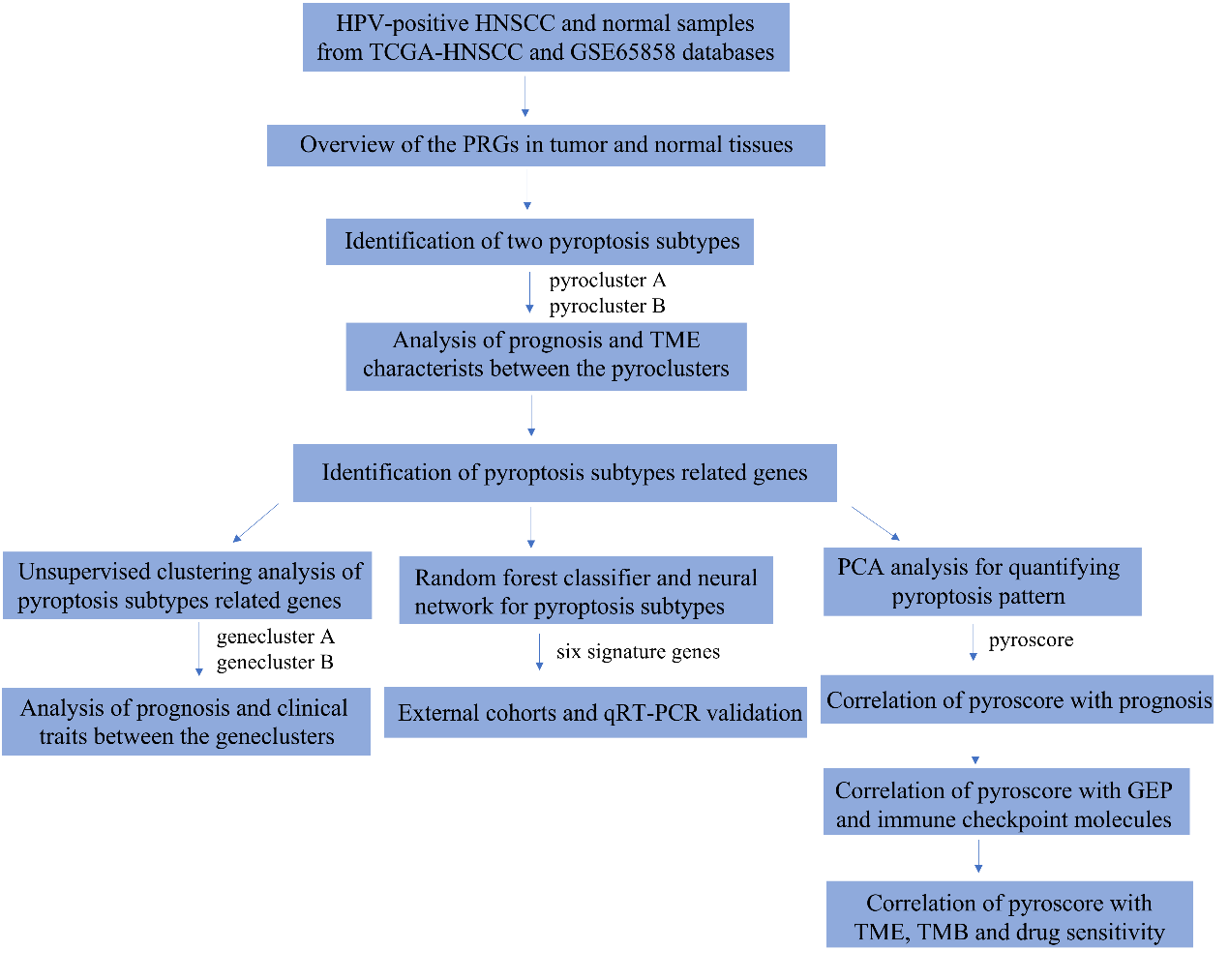


**Figure S1** The workflow of the study.


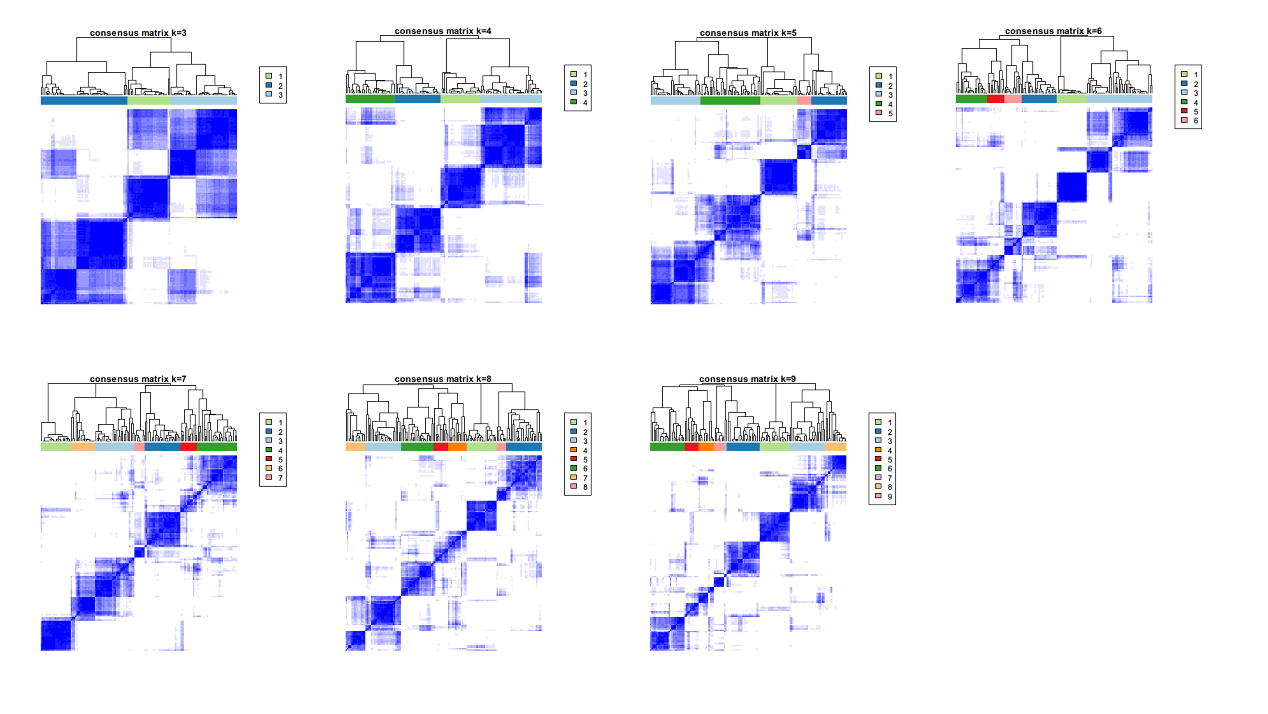


**Figure S2** Unsupervised clustering of pyroclusters and consensus matrix heatmaps for k = 3-9.

**
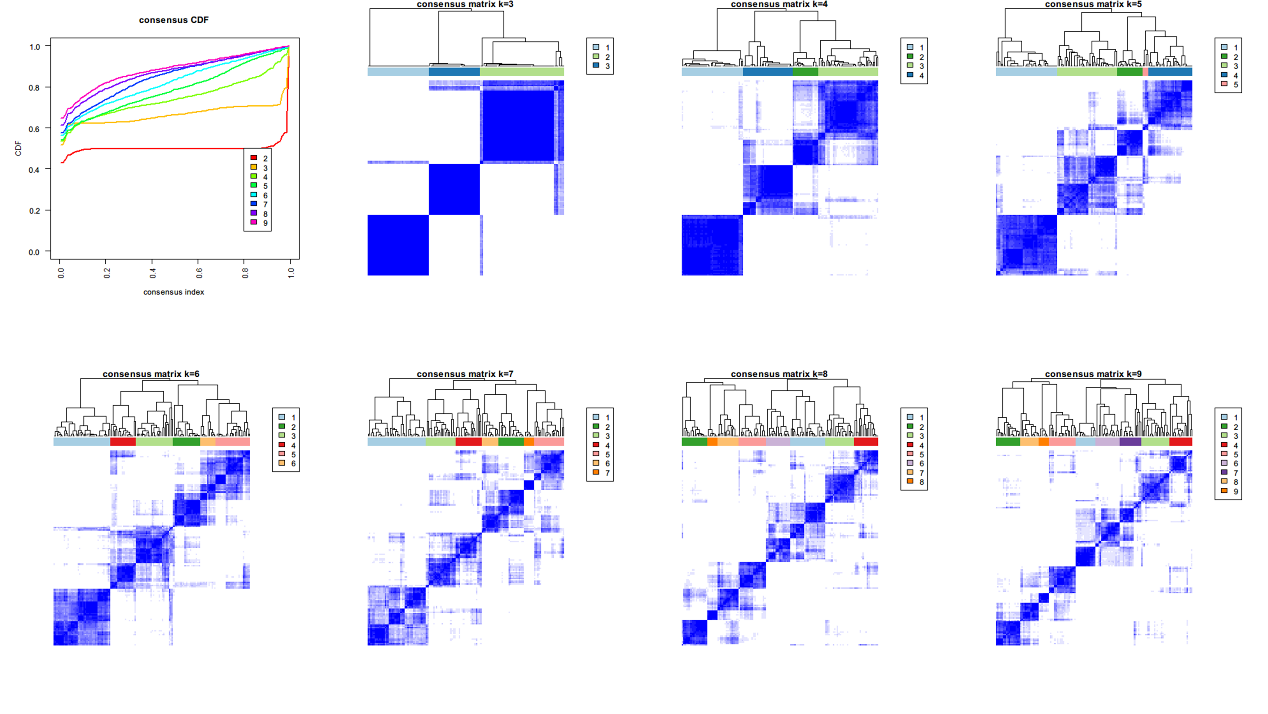
**

**Figure S3** Unsupervised clustering of pyroptosis-related geneclusters and consensus matrix heatmaps for k = 3-9.


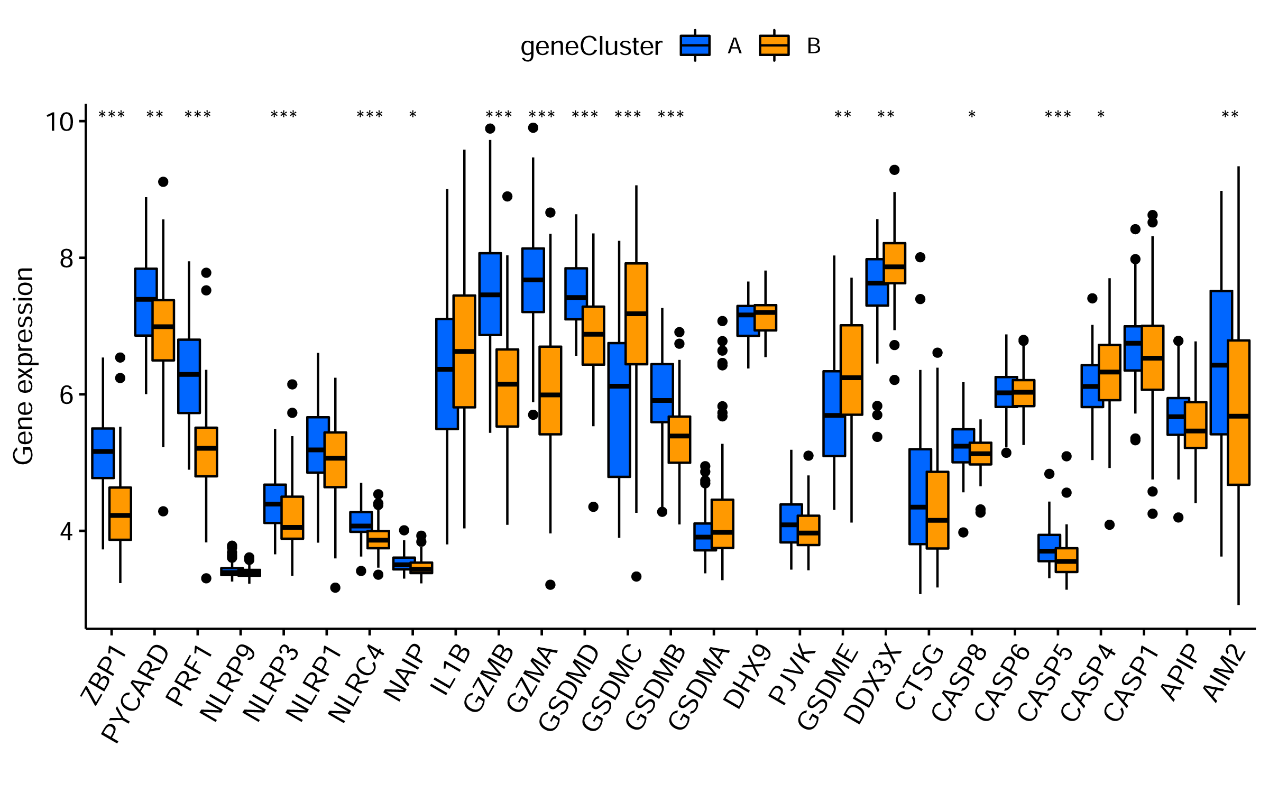


**Figure S4** Relative expression levels of 27 PRGs between the two geneclusters. **p* < 0.05, ***p* < 0.01, and ****p* < 0.001.
